# Novel Synthesis, Characterization and Amoxicillin Release Study of pH-Sensitive Nanosilica/Poly(acrylic acid) Macroporous Hydrogel with High Swelling

**DOI:** 10.3390/ma15020469

**Published:** 2022-01-08

**Authors:** Tannaz Soltanolzakerin Sorkhabi, Mehrab Fallahi Samberan, Krzysztof Adam Ostrowski, Tomasz M. Majka

**Affiliations:** 1 Department of Chemical Engineering, Ahar Branch, Islamic Azad University, Ahar P.O. Box 5451116714, Iran; tannazsoltanzakeri@gmail.com; 2 Faculty of Civil Engineering, Cracow University of Technology, Warszawska 24, 31-155 Cracow, Poland; 3 Department of Chemistry and Technology of Polymers, Faculty of Chemical Engineering and Technology, Cracow University of Technology, Warszawska 24, 31-155 Cracow, Poland; tomasz.majka@pk.edu.pl

**Keywords:** poly(acrylic acid), macroporous hydrogel, nanocomposite, high swelling ratio, drug delivery, amoxicillin

## Abstract

The effect of SiO_2_ nanoparticles on the formation of PAA (poly acrylic acid) gel structure was investigated with seeded emulsion polymerization method used to prepare SiO_2_/PAA nanoparticles. The morphologies of the nanocomposite nanoparticles were studied by transmission electron microscopy (TEM). Fourier-transform infrared (FTIR) spectroscopy results indicated that the PAA was chemically bonded to the surface of the SiO_2_ nanoparticles. Additionally, the resulting morphology of the nanocomposite nanoparticles confirmed the co-crosslinking role of the SiO_2_ nanoparticles in the formation of the 3D structure and hydrogel of PAA. SiO_2_/PAA nanocomposite hydrogels were synthesized by in situ solution polymerization with and without toluene. The morphology studies by field emission scanning electron microscopy (FESEM) showed that when the toluene was used as a pore forming agent in the polymerization process, a macroporous hydrogel structure was achieved. The pH-sensitive swelling behaviors of the nanocomposite hydrogels showed that the formation of pores in the gels structure was a dominant factor on the water absorption capacity. In the current research the absorption capacity was changed from about 500 to 4000 g water/g dry hydrogel. Finally, the macroporous nanocomposite hydrogel sample was tested as an amoxicillin release system in buffer solutions with pHs of 3, 7.2, and 9 at 37 °C. The results showed that the percentage cumulative release of amoxicillin from the hydrogels was higher in neutral and basic mediums than in the acidic medium and the amoxicillin release rate was decreased with increasing pH. Additionally, the release results were very similar to swelling results and hence amoxicillin release was a swelling controlled-release system.

## 1. Introduction

Hydrogels are a significant group of polymeric materials that are widely used in various fields of engineering and medicine such as sensors, lenses, supercapacitors, drug carriers, edible jellies, cosmetics, sanitary napkins, heavy metal ions removers, etc. [1,2,3]. Hydrogels are water insoluble, crosslinked polymers with a three-dimensional structure which can swell significantly in the water. Environmentally sensitive hydrogels as smart materials can respond to external stimuli including changes in light, temperature [4], pH [5,6], etc. Hydrogels with basic groups such as amine or acidic groups such as carboxylic acid are also called polyelectrolyte gels [7,8]. The most significant property of these gels is their high swelling degree in the aqueous media. This high swelling ratio is caused by the ions of the basic or acidic groups in the molecular structures of the polyelectrolyte gels. These anions or cations formed from basic or acid groups on the polymer chains create a repulsive force between them and so expansion of network and swelling of gels take place [9]. For example, poly acrylic acid [10,11], poly methacrylic acid [12,13], acrylamide/itaconic acid copolymer [14], acrylamide/maleic acid copolymer [15], and poly vinyl amine [16]. Polyelectrolyte gels can intelligently change their volume in response to the environmental conditions such as temperature, pH, and concentration of ions in the solution [17,18,19]. In this research, at first core shell nanocomposite nanoparticles were prepared by seeded emulsion polymerization, and then macroporous SiO_2_/PAA nanocomposite hydrogels were synthesized by in situ polymerization using acrylic acid as the monomer, methylene bisacrylamide as the crosslinker, SiO_2_ as the nanoparticles, and co-crosslinker, toluene as the pore forming agent, potassium persulphate (KPS) as the initiator, and distilled water as the solution. The effects of SiO_2_ nanoparticles and toluene on the synthesis and properties of the nanocomposite samples were investigated. It was decided to test the macroporous nanocomposite hydrogel as an amoxicillin carrier in drug delivery system due to the hydrogels excellent swelling properties.

## 2. Research Significance

Excellent properties of hydrogels such as softness, high water adsorption capacity, flexibility, biocompatibility, and non-toxic and environmentally friendly nature have made them very popular. Their high resemblance to living tissue provides many opportunities in biomedical applications. Hydrogels are currently used in a variety of biomedical applications, including drug delivery systems, scaffolds for tissue engineering, contact lenses, health products, and wound dressings. In recent decades, nanotechnology along with other scientific fields has attracted much attention and expectations. In recent decades, nanotechnology, along with other scientific fields, has attracted much attention and expectation. The combination of nanotechnology with other fields of science has attracted increasing attention during the past decades. There have been numerous approaches to incorporate nano-scale methods with conventional methods toward manufacturing improved materials. For instance, nanocomposite hydrogels are the product of the combination between two technologies, i.e., nanotechnology and biotechnology. The innovations behind the discovery of nanocomposite hydrogels are new synthesis methods and use of different types of fillers at the nanoscale for modification of conventional hydrogels. In recent years, polymer nanocomposites have attracted a lot of attention due to their potential uses in many areas [20]. SiO_2_ nanoparticles have been extensively used in nanocomposite materials due to their nanoscale size and high specific surface area [21]. SiO_2_ nanoparticles can improve various properties of polymeric nanocomposite such as mechanical property, thermal property, rheological property, and chemical property with control of these properties base on their compositions, dimensions, and structures of the core and shell [22,23,24]. Various techniques such as in situ polymerization process, melt-compounding and mechanical-mixing have been employed to synthesize polymer nanocomposites [25]. In situ polymerization can provide a type of one step polymerization, uniform dispersion of SiO_2_ nanoparticles, and enhancement of composites stability [26]. The controlled drug delivery systems deliver drug in the body deliberately at preordained rates within a calculated period of time [27]. It is worthwhile if the drug is administered into the body and accurately matches the physiological requirements. In drug release systems, the oral route is a significantly comfortable and suitable approach for the administration of drugs [28]. The pH sensitivity plays an important role in oral drug delivery as a result of the different pH in the body segments. It has been proven many times that hydrogels have the characteristics of pH sensitivity [29,30].

## 3. Experimental Section

### 3.1. Materials

Acrylic acid (AA, Merck, Darmstadt, Germany, ≥99%) and N, N′-methylenebis (acrylamide) (MBA, Sigma-Aldrich, St. Louis, MO, USA, 99%), Potassium persulfate (KPS, Panreac Química, Spain, ≥98%), sodium dodecyl sulfate (SDS, Merck, Germany, ≥90%), toluene (Sigma-Aldrich, St. Louis, MO, USA, 99.8%), silica (SiO_2_, Us Nano, Houston, TX, USA, ≥99%) nanoparticles, and distilled water were used in current research. 

### 3.2. Seeded Emulsion Polymerization of AA and SiO_2_ Nanoparticles

Seeded emulsion polymerization method was used to prepare SiO_2_/PAA core/shell nanoparticles. First, SiO_2_ nanoparticles (100 mg) were dispersed in 80 mL distilled water by ultrasonic waves and stabilized by SDS surfactant (25 mg). Then, AA monomer (1 cc, 1.05 g, 14.57 mM), MBA crosslinking agent (170 mg, 1.1 mM), and KPS (20 mg, 0.074 mM, ≅0.5% of total monomer) radical polymerization initiator were added to the prepared SiO_2_ colloidal suspension and the reaction started in the presence of nitrogen gas at 65 ± 2 °C. After 24 h, the reaction was stopped. 

### 3.3. Prepration of Core-Shell Nanoparticles

Firstly, SiO_2_ nanoparticles (100 mg) were poured into 35 mL of distilled water and sonicated for 30 min to disperse the nanoparticles, and then SDS surfactant (25 m) was added to it to stabilize the dispersed nanoparticles in the distilled water. In situ solution polymerization technique was used to prepare SiO_2_/PAA nanocomposite hydrogels. (AA monomer (5 cc, 5.25 g, 72.85 Mm) after removal of its inhibitors, (100 mg, 0.37 Mm ≅ 0.5% of total monomers) of KPS as polymerization initiator and (500 mg, 3.24 mM) of MBA crosslinking agent were added to the prepared SiO_2_ colloidal solution. Additionally, 15 mL toluene was added to the polymerization system to create macro pores in the nanocomposite hydrogels’ structure. All experiments were made in the presence of nitrogen gas at 65 ± 2 °C. After 30 min, the gel formation was observed by stopping the rotation of stir bar. This polymerization process was also repeated without silica nanoparticles and toluene.

### 3.4. Dynamic Swelling Studies

After washing with water to remove unreacted components and free oligomers or free polymer chains from the hydrogel media, the powdered nanocomposite hydrogels were added to water to study the water uptake. Samples remained in water for 24 h at room temperature and reached equilibrium swelling. All swelled samples were dried in the oven at 50 ± 2 °C. These dried hydrogels were immersed in the aqueous solutions with the different pH values (pH 2.5 ± 0.5, 10 ± 0.5) at room temperature, and the dynamic swelling experiments were conducted by measuring the water weight of samples. About 1 g of the dried samples were immersed in the aqueous buffer solution. The samples were withdrawn from the buffer solutions at various times, and then the wet weight was carefully measured as a function of time. According to the following Equation (1), the percent swelling was expressed as the percent weight ratio of the water held in the hydrogel to the dry sample at any instant during swelling.

(1) $$Swelling\;ratio\;\left(\%\right)=\frac{W_{t}-W_{d}}{W_{d}}\times 100\;\;\;$$

where *W_t_* is the weight of the swollen sample at time *t* and *W_d_* is the weight of the dry sample at time 0. 

### 3.5. Measurement of Water Retention

About 500 mg of macroporous nanocomposite hydrogel after reaching equilibrium swelling was exposed to a temperature of 50 °C. The weight loss was calculated as function of time. Equation (2) was used to determine the percentage of water retention of the samples. For accuracy, the test was repeated three times.

(2) $$Sample\;water\;retention\;\left(\%\right)=\frac{W_{t}-W_{d}}{W_{e}-W_{d}}\times 100\;\;$$

where *W_e_* is the initial weight of the equilibrium swollen macroporous nanocomposite hydrogel.

### 3.6. Loading of Amoxicillin into the Macroporous Nanocomposite Hydrogels

The loading of amoxicillin into the macroporous nanocomposite hydrogels was carried out by the swelling equilibrium method; 10 mg of dry hydrogel sample were immersed into 50 mL of 200 µg/mL amoxicillin solutions. The total daily dose of amoxicillin is 1000 mg for medication [31]. The samples were kept for 48 h in the amoxicillin solution under moderate stirring at 37 °C, after which the hydrogel samples were removed, frozen, and dried by the freeze drying method. The loaded drug amounts were determined by UV–vis spectroscopy based on the decrease in the concentration of the initial amoxicillin loaded solutions determined from the UV–vis calibration curve for amoxicillin at 276 nm. The drug loading efficiency of the hydrogel was calculated from the following Equation (3):

(3) $$Loading\;efficiency=\frac{total\;amount\;of\;amoxicillin-free\;amoxicillin}{total\;amount\;of\;amoxicillin\;}\times 100\;\;\;\;\;\;$$

### 3.7. In Vitro Drug Release

In vitro release of amoxicillin was studied at 37 °C. At specific time intervals, 3 mL of the sample solution containing released amoxicillin was taken. The absorbance of the released drug was determined by UV–vis spectrometer at 276 nm. The drug release experiments were observed in triplicate. The average drug release was calculated using the following Equation (4).

(4) $$Amoxicillin\;\left(\%\right)=\frac{released\;amoxicillin\;\;from\;macroporous\;hydrogel\;at\;\;each\;time\;of\;total\;realiesd\;time\;\;}{total\;amoxicillin\;\;in\;hydrogel}\;\times \;100$$

### 3.8. Instrumentations

The morphology of the resultant SiO_2_/PAA freeze-dried nanocomposite hydrogels were determined by Daypetronic Company using scanning electron microscopy (FESEM-Sigma VP, Zeiss, Germany) and the samples were coated with gold before FESEM characterization. The FTIR spectra of SiO_2_/PAA nanocomposite nanoparticles were recorded on Tensor 27 FTIR spectrometers (Bruker Optik GmbH, Ettlingen, Germany) using KBr discs and under strictly constant conditions in the region of 400–4000 cm^−1^. Samples were viewed using a Zeiss Leo 906 (Carl Zeiss Inc., Oberkochen, Germany) transmission electron microscope (TEM). The nanoparticle sizes of the samples were measured by a particle size analyzer using dynamic light scattering, DLS (Zetasizer Nano, Malvern Instruments Ltd., Worcestershire, UK). Amoxicillin release was studied by UV–vis spectra, which were obtained using a Perkin-Elmer Lambda 25 spectrophotometer. 

## 4. Results and Discussion

### 4.1. Effect of Silica Nanoparticles on Formation of the Gel Structure

Experimental comparison of the structure and properties of two samples, synthesized by polymerization in the presence of SiO_2_ nanoparticles and without it, indicated that the silica nanoparticles could act as a co-crosslinking agent and help 3D structure and network formation and, as a result, the hydrogel was formed. However, samples prepared without the nanoparticles did not exhibit hydrogel properties, and only solution of PAA was achieved.

### 4.2. SiO_2_/PAA Nanocomposite Nanoparticles with Core/Shell Morphology

**Figure 1:**
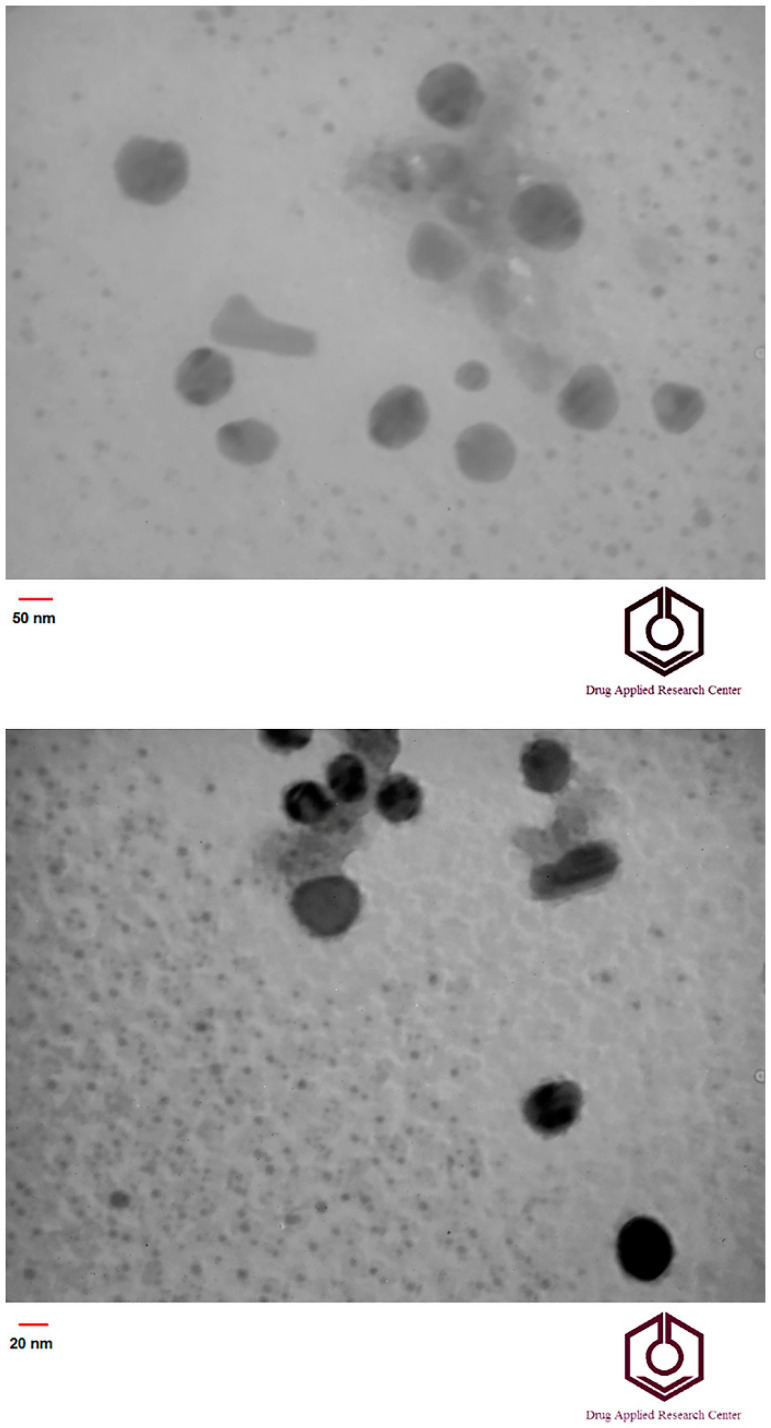
TEM images of SiO_2_/PAA core/shell nanoparticles (**upper**) and SiO_2_ nanoparticle (**lower**).

TEM images of the synthesized nanoparticles by the seeded SiO_2_ emulsion polymerization are shown in Figure 1. According to the TEM results, SiO_2_/PAA nanocomposite nanoparticles were synthesized with core/shell morphology. As shown in Figure 1, the average diameters of SiO_2_ nanoparticles and core/shell nanoparticles were about 30 and 96 nm, respectively. The formation of core/shell morphology could be attributed to the fact that the radicals are formed in water via breakdown of initiator at 65 °C to start the polymerization of monomers. The polymerization could be performed on the seed nanoparticles and in the water. The clusters, chains, and oligomers of MBA-crosslinked PAA in the aqueous phase could be collided with the surfaces of the SiO_2_ nanoparticles, leading to the reaction or nucleation and growth of a shell layer on the seeds. However, the morphology of the core/shell nanoparticles indicated that there was a good interaction between the silica nanoparticles and the poly(acrylic acid) chains. The formation of core/shell morphology confirmed the co-crosslinking role of the silica nanoparticles in the formation of the 3D structures and hydrogels. It should be noted that the amount of monomers used in the samples in Figure 1 was very low compared to other samples, and injection of more monomers into polymerization system would lead to an increase in shell thickness. With increasing shell thickness, three-dimensional structure and the hydrogel were achieved. Although the TEM results showed that the size of the nanoparticles was about 96 nm, and no particle aggregation was observed. The DLS results showed that the average nanoparticles size was 478 nm and thus was much larger than what was observed in TEM. The difference in size of the nanoparticles determined by DLS and TEM may be due to the swelling of the hydrophilic shells of nanocomposite nanoparticles in the water or the very close distance of the nanoparticles in solution.

### 4.3. Characterization of SiO_2_/PAA Nanocomposite Nanoparticles by FTIR Spectra

**Figure 2:**
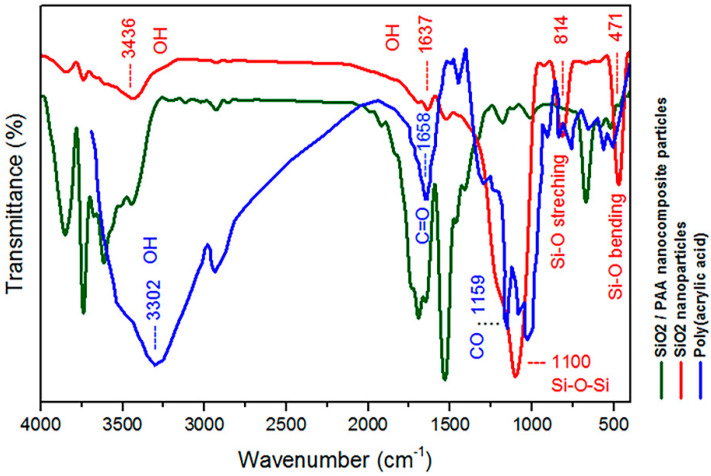
FTIR spectra of PAA, SiO_2_ nanoparticles and SiO_2_/PAA nanocomposite.

Chemical structure characterization of the PAA, SiO_2_ nanoparticles, and SiO_2_/PAA nanocomposite was achieved by FTIR, as shown in Figure 2. In this figure, the weak peaks at 3436 and 1637 cm^−1^ are due to the O–H group on the surface of SiO_2_, and the strong peak observed at 1100 cm^−1^ in the SiO_2_ spectrum is due to the Si-O-Si bonds. Additionally, the bands at 471 cm^−1^ and 814 cm^−1^ in SiO_2_ spectrum represent Si-O bending vibration and stretching vibration, respectively. The characteristic absorption peaks of pure PAA were observed at 3302 cm^−1^ for the OH hydroxyl groups and in the 1658 cm^−1^ for the C=O carbonyl groups. The peaks at about 1100 cm^−1^ are attributed to –CO in –COOH of PAA. From the spectrum of nanocomposite is obvious that characteristic absorbance peaks of SiO_2_ and PAA were shifted, and their intensity changed. This suggests that the polymer was chemically bonded to the surface of the SiO_2_ nanoparticles.

### 4.4. Creation of Macro Pores in the Hydrogel Structure and Microstructure Morphology of the SiO_2_/PAA Macroporous Hydrogels

**Figure 3:**
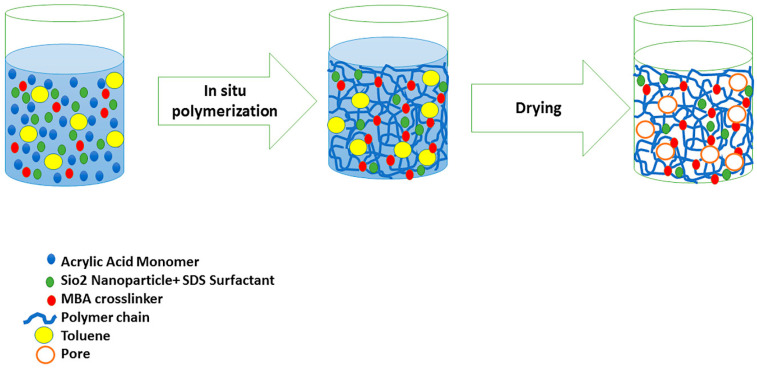
Schematic synthesis method of macroporous SiO_2_/PAA nanocomposite hydrogel and the proposed mechanism for macro pore formation.

**Figure 4:**
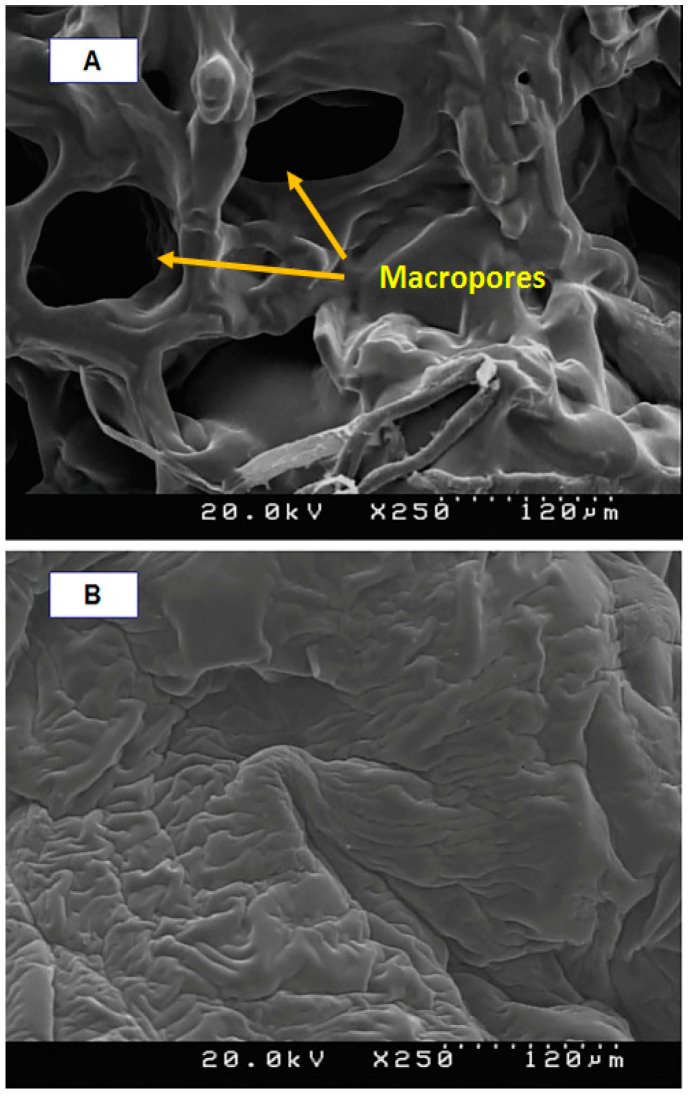
FESEM micrographs of air-dried samples synthesized with toluene (**A**) and without toluene (**B**).

**Figure 5:**
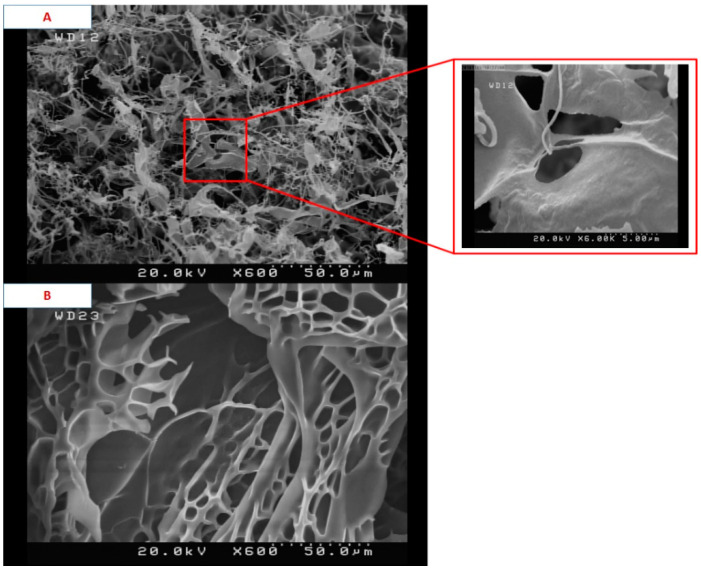
FESEM micrographs of freeze-dried samples synthesized with toluene (**A**) and without toluene (**B**).

Details of the macroporous nanocomposite hydrogel preparation are schematically shown in Figure 3. As shown in this figure, the synthesis was done in the presence of toluene that finally resulted in three-dimensional macro pores formation in the structure of the hydrogel by removing it from the final product. The presence of macropores in hydrogel structure resulted in improved swelling properties and caused absorption of very large amounts of water. In general, the microstructure morphologies of polymeric hydrogels are studied by FESEM. The FESEM micrographs of the air-dried samples synthesized with and without toluene are shown in Figure 4. It can be seen that the morphologies of the air-dried samples prepared with and without toluene were very different at the same magnification, and no macro pores were observed in the sample synthesized without toluene. However, the SiO_2_/PAA nanocomposite hydrogels synthesized in the presence of toluene had interconnected macroporous network structure with macropores of diameter up to tens of microns. The existence of macropores can be related to the vaporization and removing of the toluene from the hydrogel structure rather than phase separation and gel formation during the polymerization. In fact, the macroporous structure increased the specific surface area, resulting in good interaction between matrix and improvement of the water absorption and swelling properties of the hydrogel. The swelled macroporous samples were also dried using a freeze-drying technique to retain the original pore structure and then observed in the microscope. The FESEM micrograph of freeze-dried samples are shown in Figure 5. These micrographs show the high porosity and three-dimensional interconnected microstructures similar to other reported polymeric hydrogel structures. The inner interwoven structure and interconnectivity of the pores in the hydrogel could be assigned to the crosslinking of polymeric chains. The observed high porosity in the micrographs which arose from evaporation of the water absorbed by the hydrophilic carboxyl groups of PAA during the freeze-drying process. According to the FESEM results, synthesized macroporous nanocomposite hydrogel in the current research had fibrous morphology, and the macropores were not observable in the microscope due to their large size after swelling and freeze-drying. It is worth mentioning that in the morphology of this sample, with more magnification, some thin layers were also observed, which might be due to wall formation in interface of toluene and solution during the polymerization process. 

### 4.5. pH-Sensitive Swelling Behaviors of SiO_2_/PAA Nanocomposite Hydrogels

**Figure 6:**
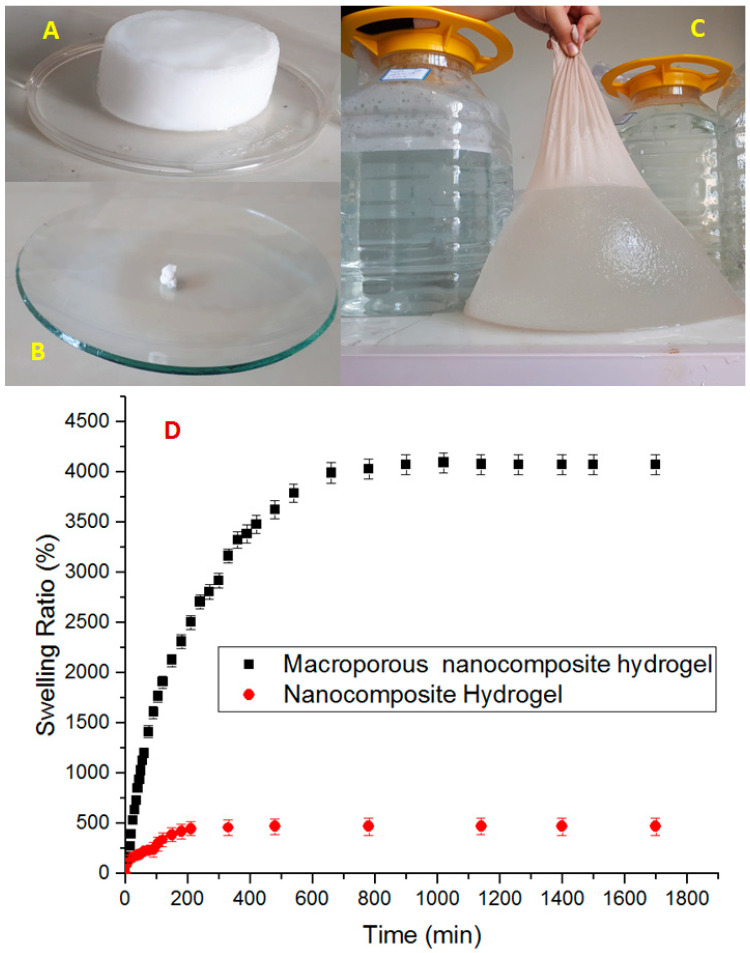
Pictures of macroporous nanocomposite hydrogel: after synthesis (**A**), dried and grinded (**B**), swollen 4000-fold from its own dry weight (**C**), and swelling ratio of nanocomposite hydrogel and macroporous nanocomposite hydrogel (**D**).

It is well established that the porosity of the hydrogel has a great effect on its water absorbency and it is the most important factor for many applications [32]. Swelling of polyelectrolyte gels such as PAA is the result of balance between the repulsive forces of polymer chains caused by ionic groups and the constraints imposed by crosslinked structure. When the pH of solution is raised above the dissociation constant (pKa), Polyacid hydrogels swell sharply. The pKa value of the carboxylic acid ionic groups of PAA is about 4.5 [33]. The results of the swelling ratios for samples at pH 10 are shown in Figure 6, and no significant swelling was observed at pH 2. At high pH value (pH > pKa) the carboxylic groups (-COOH) of PAA chains changed into carboxylate ions ($–\mathrm{COO}{}^{-}$) due to deprotonation. The carboxylate ions caused the electrostatic repulsion to increase, and thus increased the osmotic pressure and swelling ratio. In fact, the electrostatic repulsion led to more space being available for water adsorption in the hydrogel structure and caused the network to expand [34]. At low pH (pH < pKa), the –COO^−^ groups of the hydrogel were protonated and reduced the main anion–anion (COO^−^–COO^−^) repulsive force, the osmotic pressure became almost zero, being the reason why no swelling was observed at pH 2. Thus, the samples synthesized in this research exhibited a pH-sensitive swelling behavior. Figure 6 also illustrated the important effect of toluene as a pore forming agent on the swelling percentage. It can be seen that the macropourous sample possessed a much higher swelling ratio than the hydrogel prepared without toluene. From the FESEM micrographs, it can be seen that when the toluene was used as a pore forming agent in the polymerization system, a macroporous structure hydrogel was achieved. It is supposed that these macropores are regions of water permeation and interaction sites. The macroporous architectures of our nanocomposite hydrogels provide large specific surface area, resulting in better water–hydrogel interaction, easier water absorption, and in larger equilibrated swelling ratios. Therefore, the formation of pores in the gels structure is a dominant factor that determines the swelling properties, and in our current research, the absorption capacities were improved from about 500 to 4000 g water/g dry hydrogel.

### 4.6. Water Retention of the Macropouros Nanocomposite Hydrogels

**Figure 7:**
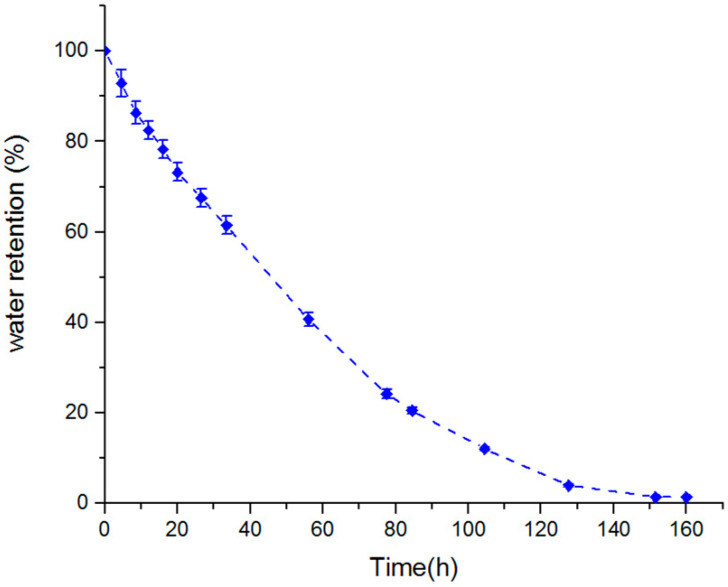
Water retention of swollen macropouros nanocomposite hydrogel at 50 °C.

Figure 7 shows the water retention of the macroporous nanocomposite hydrogel as a function of time for a temperature of 50 °C. The sample in this temperature experienced an 80% loss of water after 80 h, and it took about 160 h to release all the water content. After 80 h, the relative content of strongly absorbed water in the sample was raised and, hence, the rate of dehydration became slow. The water retention behavior of a superabsorbent can be related to the interaction of H-bonding and Van der Waals forces between the solvent molecules and the superabsorbent [35]. The carboxylate and hydroxyl groups in the PAA hydrogel were responsible for considerable interaction and, hence, enhanced the water retention power. The reason for doing the water retention test at a high temperature was that the water release from the composite structure and deswelling process at a high temperature is caused by the weakening and breaking both H-bonding and Van der Waals between molecules of water and hydrogel chains, so that the water was easily released.

### 4.7. Amoxicillin Release Studies

**Figure 8:**
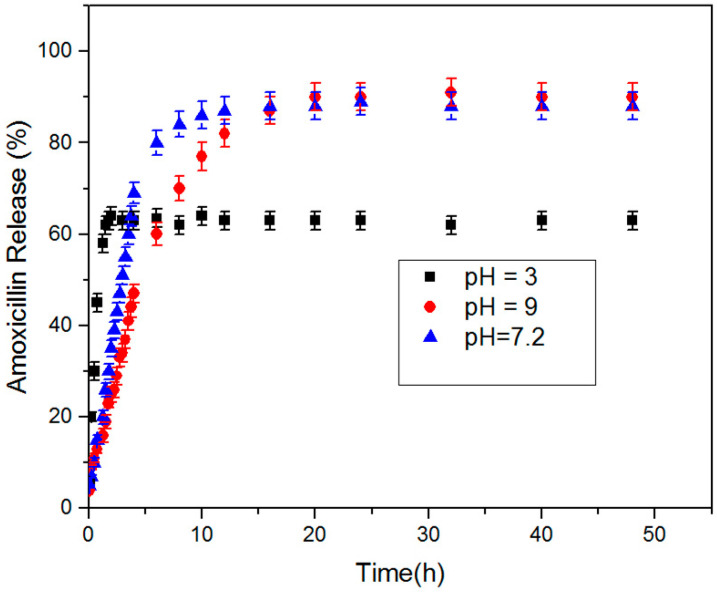
Amoxicillin release behavior of macroporous nanocomposite hydrogel at 37 °C and pH values of 3, 7.2, and 9.

Figure 8 shows the release profiles of amoxicillin from the prepared macroporous nanocomposite hydrogel as a carrier in buffer solutions with pHs of 3 (acid environment similar to stomach), 7.2 (amoxicillin is mostly absorbed in the small intestine where the pH is neutral), and 9 (basic environment) at 37 °C. The concentration of released amoxicillin was calculated by a UV spectrophotometer at selected time intervals. As shown in Figure 8, the macroporous nanocomposite hydrogel, as a smart material, has different swelling behaviors at the different pH and have shown different releases of the amoxicillin molecules through the hydrogel networks. The hydrogels networks shrank at low pH and resulted in the faster release of the amoxicillin through a squeezing action [36]. This feature has been used for “on/off” drug delivery systems. The percentage cumulative release of amoxicillin from the hydrogels was high in neutral and basic mediums compared with acidic medium. Additionally, the change of pH affected the amoxicillin release rate and it decreased with increasing pH. As shown in Figure 6 and Figure 8, the release results were very similar to swelling results and, hence, amoxicillin release can be considered a swelling controlled-release system. The charge repulsion of chemical groups on the PAA chains increase when the pH of the medium raise above the pK of the carboxyl groups of PAA, and so hydrophilicity, and as a result the amoxicillin release has increased.

## 5. Conclusions

To date, polymeric superabsorbent materials with such a high swelling ratio (more than 3000 g g^−1^) have rarely been reported. Nanocomposite superabsorbents are the new types of polymeric networks that swell markedly, but do not dissolve in aqueous media. The ability of superabsorbents to absorb water arises from hydrophilic functional groups such as -NH_2_, -COOH, -OH, -CONH, -CONH-, and -SO_3_H attached to the polymeric backbone. Due to its superior water absorption property, superabsorbent hydrogels are applied in various industries, such as soil amendment, the construction industry, responsive materials, and wastewater treatment. Especially in agriculture and forestry, superabsorbent hydrogels can be employed as water-retaining agents to improve soil water retention. According to the TEM results, SiO_2_/PAA nanocomposite nanoparticles with complete core/shell morphology synthesized by seeded emulsion polymerization. The co-crosslinking role of the silica nanoparticles in the formation of the 3D structures and the hydrogel was confirmed by FTIR analysis. Macroporous SiO_2_/PAA nanocomposite hydrogels were synthesized by using toluene in the in situ solution polymerization and the macroporous architectures of nanocomposite hydrogel was characterized by FESEM images. The macroporosity of the structure improved its water absorption capacities from about 500 to 4000 g water/g dry hydrogel. Superabsorbent hydrogels are a new type of polymeric material with a 3D network structure that can absorb a lot of water. The macroporous morphology of the nanocomposite hydrogels provided large specific surface areas, resulting in better water–hydrogel interaction, easier water absorption, and larger equilibrated swelling ratio. The water retention of macroporous nanocomposite sample was studied as a function of time at 50 °C. The sample at 50 °C experienced an 80% loss of water after 80 h, and it took about 160 h to release all the water content. The macroporous samples were tested as an amoxicillin release system in buffer solutions with pHs 3, 7.2, and 9 at 37 °C. The percentage cumulative release of amoxicillin from the hydrogels was higher in the basic and neutral mediums than in the acidic medium. As the pH of the medium was increased above the pK of the carboxyl groups of the polyanions, charge repulsion increased due to the hydrophilicity increasing and, as a result, amoxicillin release was increased.

## Data Availability

The data presented in this article are available within the article.

## References

[1] Khan S.A., Shah L.A., Shah M., Jamil I. (2021). Engineering of 3D polymer network hydrogels for biomedical applications: A review. Polym. Bull..

[2] Onaciu A., Munteanu R.A., Moldovan A.I., Moldovan C.S., Berindan-Neagoe I. (2019). Hydrogels Based Drug Delivery Synthesis, Characterization and Administration. Pharmaceutics.

[3] Feksa L.R., Troian E.A., Muller C.D., Viegas F., Machado A.B., Rech V.C. (2018). Hydrogels for biomedical applications. Nanostruct. Eng. Cells Tissues Organs.

[4] Razavi B., Abdollahi A., Roghani-Mamaqani H., Salami-Kalajahi M. (2019). Light-, temperature-, and pH-responsive micellar assemblies of spiropyran-initiated amphiphilic block copolymers: Kinetics of phototropism, responsiveness, and smart drug delivery. Mater. Sci. Eng. C.

[5] Hu X., Wei W., Qi X., Yu H., Feng L., Li J., Wang S., Zhang J., Dong W. (2015). Preparation and characterization of a novel pH-sensitive Salecan-g-poly(acrylic acid) hydrogel for controlled release of doxorubicin. J. Mater. Chem. B.

[6] Kang X.J., Dai Y.L., Ma P.A., Yang D.M., Li C.X., Hou Z.Y., Cheng Z.Y., Lin J. (2012). Poly(acrylic acid)-modified Fe_3_O_4_ microspheres for magnetic targeted and pH-triggered anticancer drug delivery. Chem. Eur. J..

[7] Horkay F. (2021). Polyelectrolyte Gels: A Unique Class of Soft Materials. Gels.

[8] Skouri R., Schosseler F., Munch J.P., Candau S.J. (1995). Swelling and elastic properties of polyelectrolyte gels. Macromolecules.

[9] Kwon H.J., Osada Y., Gong J.P. (2006). Polyelectrolyte Gels-Fundamentals and Applications. Polym. J..

[10] Jeong J., Baik J., An S., Jeong S., Lee J., Lim Y., Park J. (2018). Development and Characterization of Cross-Linked Poly(acrylic acid) Hydrogel Containing Drug by Radiation-Based Techniques. Preprints.

[11] Kazanskii K.S., Dubrovskii S.A. (1992). Chemistry and Physics of “Agricultural” Hydrogels. Adv. Polym. Sci..

[12] Lumbreras-Aguayo M.J.A., Meléndez-Ortiz H.I., Puente-Urbina B., Alvarado-Canché C., Ledezma A., Romero-García J., Betancourt-Galindo R. (2019). Poly(methacrylic acid)-modified medical cotton gauzes with antimicrobial and drug delivery properties for their use as wound dressings. Carbohydr. Polym..

[13] Bajpai S.K., Chand N., Mahendra M. (2013). In situ formation of silver nanoparticles in poly(methacrylic acid) hydrogel for antibacterial applications. Polym. Eng. Sci..

[14] Angar N.E., Aliouche D. (2016). Rheological behavior and reversible swelling of pH sensitive poly(acrylamide-co-itaconic acid) hydrogels. Polym. Sci. Ser. A.

[15] Bajpai S.K. (2001). Swelling–deswelling behavior of poly(acrylamide-co-maleic acid) hydrogels. J. Appl. Polym. Sci..

[16] Mendoza-Payan J.G., Flores-Gallardo S., Marquez-Lucero A. (2010). Preparation and electrical characterization of poly(vinyl amine) hydrogels, crosslinked with Cu (II) ions, and evaluation of their potentiality as water sensor material. J. Appl. Polym. Sci..

[17] Rizwan M., Yahya R., Hassan A., Yar M., Azzahari A.D., Selvanathan V., Sonsudin F., Abouloula C.N. (2017). pH Sensitive Hydrogels in Drug Delivery: Brief History, Properties, Swelling, and Release Mechanism, Material Selection and Applications. Polymers.

[18] Anuraag B., Bratlie K.M. (2018). Collagen organization deposited by fibroblasts encapsulated in pH responsive methacrylated alginate hydrogels. J. Biomed. Mater. Res. Part A.

[19] Didem A., Alemdar N. (2018). Development of pH-responsive chitosan-based hydrogel modified with bone ash for controlled release of amoxicillin. Carbohydr. Polym..

[20] Crosby A.J., Lee J.Y. (2007). Polymer Nanocomposites: The “Nano” Effect on Mechanical Properties. Polym. Rev..

[21] Liu Z., Du J., Tan Y., Cao L., Xu S., Huang J. (2018). Strengthening Network of Polyacrylic Acid/Silica Nanocomposite Hydrogels. Polym. Compos..

[22] Guo M., Ming J., Stergios P., Wei Y., Zhou C. (2008). Supramolecular hydrogels made of end-functionalized low-molecular-weight PEG and α-Cyclodextrin and their hybridization with SiO_2_ nanoparticles through host−guest interaction. Macromolecules.

[23] Lee D.W., Yoo B.R. (2016). Advanced silica/polymer composites: Materials and applications. J. Ind. Eng. Chem..

[24] Chowdhury P., Saha S.K., Guha A., Saha S.K. (2012). Chemical and biochemical activities of sonochemically synthesized poly(N-isopropyl acrylamide)/silica nanocomposite. Appl. Surf. Sci..

[25] Puggal S., Dhall N., Singh N., Litt M.S. (2016). A Review on Polymer Nanocomposites: Synthesis, Characterization and Mechanical Properties. Indian J. Sci. Technol..

[26] Liu H., Wang T., Wang Q. (2012). In situ synthesis and properties of PMR PI/SiO_2_ nanocomposites. J. Appl. Polym. Sci..

[27] Nadeem M., Ahmad M., Akhtar M.S., Shaari A., Riaz S., Naseem S., Saeed M.A. (2016). Magnetic Properties of Polyvinyl Alcohol and Doxorubicine Loaded Iron Oxide Nanoparticles for Anticancer Drug Delivery Applications. PLoS ONE.

[28] Lipinski C.A., Lombardo F., Dominy B.W., Feeney P.J. (2012). Experimental and computational approaches to estimate solubility and permeability in drug discovery and development settings. Adv. Drug Deliv. Rev..

[29] Malik N.S., Ahmad M., Minhas U.M. (2017). Cross-linked β-cyclodextrin and carboxymethyl cellulose hydrogels for controlled drug delivery of acyclovir. PLoS ONE.

[30] Li J., Mooney D.J. (2016). Designing hydrogels for controlled drug delivery. Nat. Rev. Mater..

[31] Rodríguez-Félix D.E., Pérez-Martínez C.J., Castillo-Ortega M.M., Pérez-Tello JRomero-García A., Ledezma-Pérez S., del Castillo-Castro T., Rodríguez-Félix F. (2012). pH- and temperature-sensitive semi-interpenetrating network hydrogels composed of poly(acrylamide) and poly(c-glutamic acid) as amoxicillin controlled-release system. Polym. Bull..

[32] Ahmed E.M. (2015). Preparation, characterization, and applications: A review. J. Adv. Res..

[33] Fallahi S.M., Salami K.M., Dehghani E., Abbasi F. (2019). Investigating Janus morphology development of poly(acrylic acid)/poly(2-(dimethylamino)ethyl methacrylate) composite particles: An experimental study and mathematical modeling of DOX release. Microchem. J..

[34] Gupta P., Vermani K., Garg S. (2002). Hydrogels: From controlled release to pH-responsive drug delivery. Drug Discov. Today.

[35] Wu F., Zhang Y., Liu L., Yao J. (2012). Synthesis and characterization of a novel cellulose-g-poly(acrylic acid-co-acrylamide) superabsorbent composite based on flax yarn waste. Carbohydr. Polym..

[36] Fallahi S.M., Salami K.M., Dehghani E., Abbasi F. (2018). Investigation of different core-shell toward Janus morphologies by variation of surfactant and feeding composition: A study on the kinetics of DOX release. Colloids Surf. B Biointerfaces.

